# Magnetic resonance spectroscopy associations with clinicopathologic features of estrogen-dependent endometrial cancer

**DOI:** 10.1186/s12880-022-00856-9

**Published:** 2022-07-18

**Authors:** Jie Zhang, Qingwei Liu, Jie Li, Zhiling Liu, Ximing Wang, Na Li, Zhaoqin Huang, Han Xu

**Affiliations:** 1 Department of Radiology, Shandong Provincial Hospital Affiliated to Shandong First Medical University, No. 324, Jingwu Road, Jinan, 250021 Shandong China; 2 Special Inspection Department, Taian City Central Hospital Branch, No. 336, Wanguan Road, Taian, 271000 Shandong China; 3 Department of Gynecology, Shandong Provincial Hospital Affiliated to Shandong First Medical University, No. 324, Jingwu Road, Jinan, 250021 Shandong China

**Keywords:** Type I endometrial cancer, Ki-67, Local invasiveness, Magnetic resonance spectroscopy

## Abstract

**Background:**

We studied the magnetic resonance spectroscopy (MRS) associations with clinicopathologic features of estrogen-dependent endometrial cancer (type I EC).

**Methods:**

Totally 45 patients with type I EC who underwent preoperative multi-voxel MRS at 3.0 T were enrolled. The mean ratio of the Cho peak integral to the unsuppressed water peak integral (Cho/water) of the tumor was calculated. The Cho/water and apparent diffusion coefficient (ADC) of type I EC with and without local invasion, as well as with different levels of Ki-67 staining index (SI) (≤ 40% and > 40%), were compared. Correlation test was used to examine the relationship of Cho/water, as well as mean ADC, with Ki-67 SI, tumor stage, and tumor grade.

**Results:**

The mean Cho/water of EC with Ki-67 SI ≤ 40% (2.28 ± 0.93) × 10^−3^ was lower than that with Ki-67 SI > 40% (4.08 ± 1.00) × 10^−3^ (*P* < 0.001). The mean Cho/water of EC with deep and superficial myometrial invasion was (3.41 ± 1.26) × 10^−3^ and (2.43 ± 1.11) × 10^−3^, respectively (*P* = 0.011). There was no significant difference in Cho/water between type I EC with and without cervical invasioin ([2.68 ± 1.00] × 10^−3^ and [2.77 ± 1.28] × 10^−3^, *P* = 0.866). The mean Cho/water of type I EC with and without lymph node metastasis was (4.02 ± 1.90) × 10^−3^ and (2.60 ± 1.06) × 10^−3^, respectively (*P* = 0.014). The Cho/water was positively correlated with the Ki-67 SI (r = 0.701, *P* < 0.001). There were no significant differences in ADC among groups (all *P* > 0.05).

**Conclusion:**

MRS is helpful for preoperative assessment of clinicopathological features of type I EC.

## Background

Endometrial cancer (EC) is one of the most common female genital tract cancers, which could be divided into the estrogen-dependent (type I) and non-estrogen-dependent (type II) EC types. Type I EC only contains endometrioid adenocarcinoma, which is the most common pathological type. Type I EC occurs in about 80% of the EC cases. Type II EC is more rarely seen, which generally contains multiple subtypes of EC, and is closely related to lymphatic metastasis and poor prognosis. Type II EC accouts for the remaining 20% of all the EC cases [1].

Precise assessment of EC aggressiveness before treatment may contribute to personalized treatment and prognosis prediction. Tumor proliferation activity is also an indicator to evaluate the EC aggressiveness, in addition to tumor pathological type, grade, stage and size [2]. Ki-67, a proliferation-related nuclear antigen, is expressed in all cycling cells, except for the resting cells in the G0 phase. Ki-67 staining index (SI) reflects the tissue proliferation activity. It has been suggested that the Ki-67 level in the type I EC tissue has been shown to be higher than that in the endometrial polyps [3]. High expression level of Ki-67 has been correlated with relatively higher incidence of distant recurrence [4].

As a non-invasive examination, magnetic resonance spectroscopy (MRS) is a method to obtain biochemical information from tissues. It is reported that MRS can differentiate between malignant and benign lesions of the uterus [5]. In our previous studies, MRS helped to differentiate EC from benign lesions in endometria or in submucosa, and also differentiated the type II from type I EC [6, 7]. Choline-containing compounds (Cho) are marker of active tumors, which is increased in actively proliferating tissues. The ratio of Cho peak integral and water peak integral (Cho/water) represents the concentration of Cho to a certain extent. Preoperative diagnostic curettage pathology is the main method for the diagnosis of EC. However, the specimens are limited and cannot reflect the overall pathological characteristics of the tumor. Although MRS only plays an auxiliary role, multi-voxel MRS can cover the whole tumor and reflect the metabolic characteristics of the whole tumor.

In this study, the relationship between Cho/Water and clinicopathologic features, including Ki-67 SI, of type I EC was investigated. Our findings may help to preoperatively understand the clinicopathologic features of EC to some extent.

## Methods

### Study subjects

This is a retrospective analysis. Patients with type I EC, who received hysterectomy from March 2012 to May 2014, were included in this study. The time interval between MR imaging and surgery was 1–10 days (Median, 4 days). These patients underwent total hysterectomy with bilateral salpingo-oophorectomy and pelvic lymph node dissection. The International Federation of Gynecology and Obstetrics (FIGO) stage, local invasion (myometrial invasion, cervical invasion and lymph node metastasis), grade, and size (maximum diameter) were determined by an experienced pathologist. The tumor stage was determined according to the FIGO revised staging criteria in 2014 [8].

Inclusion criteria were as follows: 1) patients with type I EC confirmed by surgical pathology (estrogen receptor, positive); 2) patients who underwent the MRS; and 3) patients with complete information, including tumor FIGO stage, grade, size and Ki-67 SI. Exclusion criteria: Subjects who had no satisfactory MRS voxel in lesions were excluded. Informed consent was waived by the ethics review board of Shandong Provincial Hospital Affiliated to Shandong First Medical University because this is a retrospective analysis. All methods were performed in accordance with the Declaration of Helsinki and the study was approved by the ethics review board of Shandong Provincial Hospital Affiliated to Shandong First Medical University (Approval No.: SWYX2020-051).

### MR imaging

MR examination was performed with a 3.0-T system (Magnetom Verio, Siemens, Germany), equipped with an eight-channel pelvic phased-array surface coil and integrated spine coils. Before MR imaging, patients were fasted for 4 h. Raceanisodamine hydrochloride injection (Minsheng Pharma., Hangzhou, China) was administered before image acquisition to reduce bowel motion. The bladder was partially filled.

**Fig. 1:**
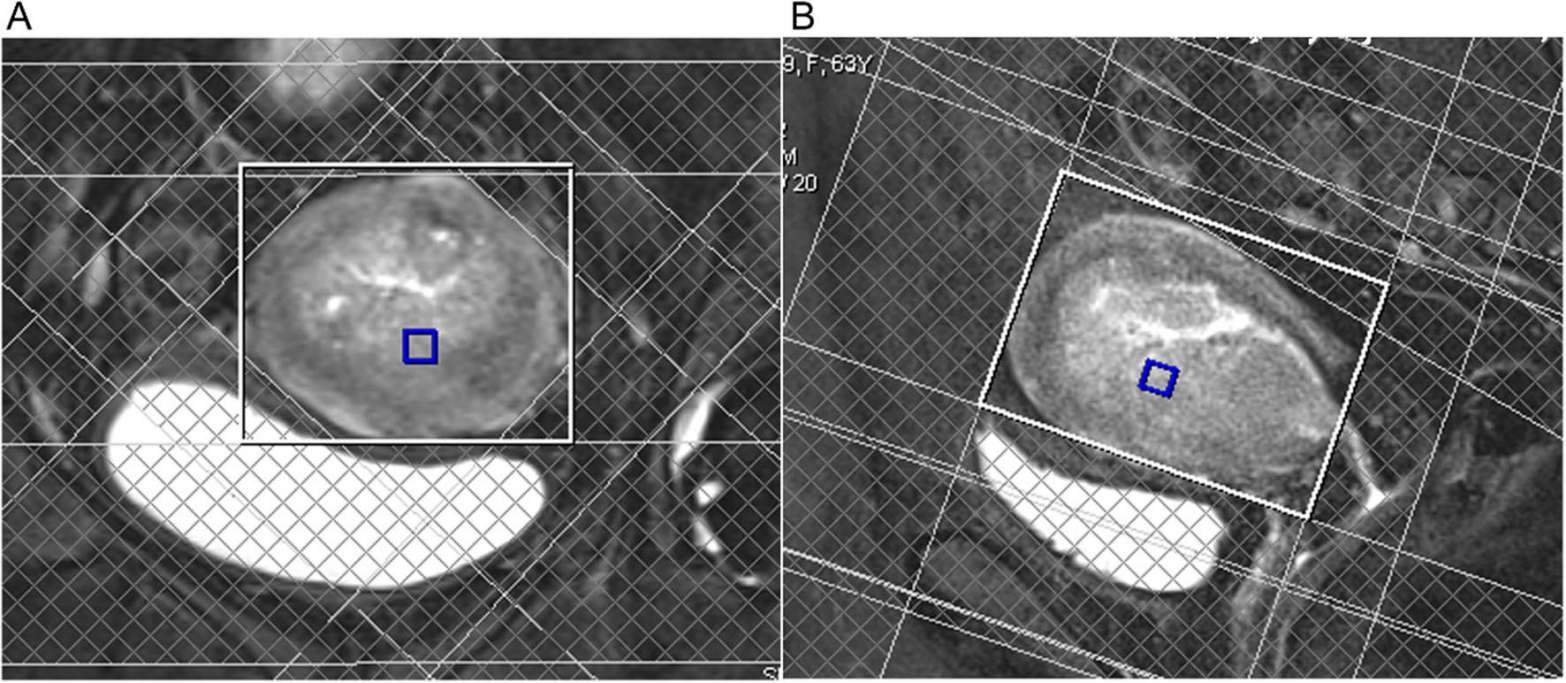
Eight saturation bands were positioned around the uterus and lesion. The white box indicates the volume of interest. **A** Six saturated bands were placed around the uterus. **B** the other two saturated bands were placed above the fundus and below the lesion

MR imaging included the 3D multi-voxel 1H MRS besides the routine sequences, i.e., the T2-weighted (T2W) imaging, diffusion-weighted (DW) imaging (DWI), and dynamic contrast enhanced (DCE) imaging. Conventional MR imaging parameters were shown in Table 1. MRS (with the parameters of TR, 750 ms; TE, 145 ms; flip angle, 90°; vector size, 512; and bandwidth, 1250 Hz) was performed with 3D chemical shift imaging techniques based on point-resolved spectroscopic sequence. A weighted elliptical K-Space acquisition mode was used to save the scan time, and a Hamming filter with a width of 100% (relative to the dimension of the K-Space) was applied to the K-Space data to suppress the contamination from the adjacent voxel due to the point-spread function. For Choline acquisition, fat and water were simultaneously suppressed by using the MEGA pulses [9], while for water signal acquisition, MEGA pulses with only fat suppression were applied. Unsuppressed and suppressed water MRS shared identical imaging parameters except the average number of acquisitions, which were 6 for water suppression spectra and 2 for unsuppressed water spectra. Eight saturation bands were positioned around the uterus and lesion, to suppress the signal contamination from the extrauterine lipids or the bladder (Fig. 1). The field of view (FOV) was 84 mm × 84 mm. The matrix was 12 × 12. The voxel size was 7 mm × 7 mm × 7 mm. MRS data were overlaid on the corresponding axial, sagittal and coronal T2W images. The acquisition of MRS data took about 16 min. Anatomy images, including axial, sagittal and coronal T2W images and DCE images, were used as reference images to select the voxels of interest in the solid part of lesions, avoiding cystic or necrotic areas. Therefore, although the FOV covered the lesion, the effective voxels were selected in the solid part of the tumor.

**Table 1:** Parameters of conventional magnetic resonance imaging sequences

Sequences	TR (ms)	TE (ms)	ST (mm)	Average	FOV (cm^2^)	Matrix
Axial T2W	3110	101	3–4.5	2	20 × 20	320 × 256
Coronal T2W	3350	97	3–4	2	20 × 20	320 × 256
Sagittal T2W	2950	101	3–4	2	20 × 20	320 × 310
DWI^a^	6200	63	3–4.5	6	20 × 20	160 × 120
T1W-DCE (VIBE)	5.21	1.8	3–4	1	26 × 26	224 × 161

*TR* Repetition time, *TE* Echo time, *ST* Slice thickness, *FOV* Field of view, *DCE* Dynamic contrast enhancement imaging, *VIBE* Volume interpolated body examination, *DWI* Diffusion-weighted imaging

^a^b value = 0, 100, 400, and 800 s/mm^2^

### MRS data analysis

MRS processing was performed with the jMRUI v.5.2 software (http://sermn02.uab.es/mrui). The Cho and water peaks were quantified. On the spectrum of suppressed water, the Cho peak was detected at 3.2 ppm. On the spectrum of unsuppressed water, the water peak was detected at 4.7 ppm. The spectra needed to be pre-processed before Cho peak quantification, as follows: filtering with “Lorentzian” 5 Hz, further water suppression and Fourier transformation. If there was phase offset for the Cho peak, the phase correction was performed. The same preprocessing was performed for the water peak, except for further water suppression. The time domain fitting algorithm AMARES (Advanced Method for Accurate, Robust and Efficient Spectral fitting) was used to quantify the water peak and Cho peak. The amplitude standard deviation (i.e., the Cramér-Rao standard deviation [CRSD]) of the metabolites could be obtained from the AMARES fitting algorithm. The CRSD could be used to measure the accuracy of metabolite peak fitting, which reflected the signal-to-noise ratio (SNR). The relative CRSD of metabolite was calculated by the CRSD/amplitude, which was inversely related to SNR. The availability of the spectrum was determined by a radiologist and a spectroscopist in consensus (both blinded to the patients’ clinical information), according to the correct positions of the Cho and water peaks, relatively stable baseline and absence of large lipid signals. Any spectrum with a relative metabolite CRSD greater than 20% or full width at half maximum (FWHM) greater than 15 Hz was excluded. There were a total of 2364 voxels of type I EC (median, 32; range, 1–273 per patient) included. The radiologist also interpreted the conventional MR images.

The Cho/water was the statistical unit herein (Eq. 1). In Eq. 1, Cho_*i*_ was the *i*^th^ voxel from suppressed water; water_*i*_ was the *i*^th^ voxel from unsuppressed water; *n* was the total number of included voxels for a patient. The Cho/water reflected the Cho concentration in tissues.

1 $${\text{Cho/water}} = (\sum\limits_{i = 1}^{n} {{\text{Cho}}_{i} /{\text{water}}_{i} } )/n$$

### DWI data analysis

**Fig. 2:**
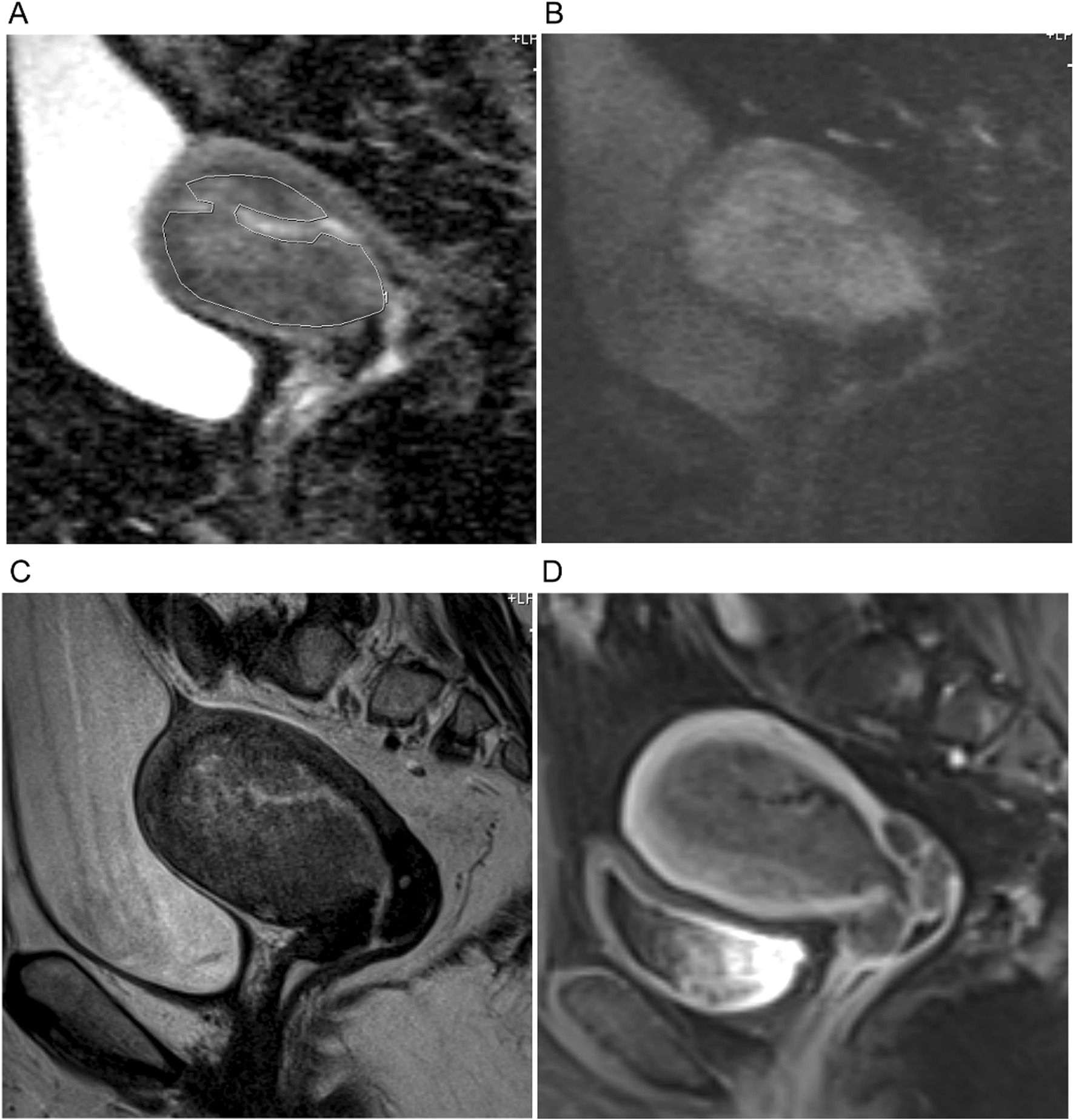
The regions of interest (ROI) were placed on ADC map (**A**) in the tumor, avoiding cystic and necrotic areas with DW image (b = 800 s/mm^2^) (**B**), T2W images (**C**) and DCE image (**D**) as reference images

ADC maps were automatically generated from DW images (b = 0, 100, 400 and 800 s/mm^2^). The regions of interest (ROI) were placed on ADC map in the tumor by two radiologists independently, according to lesion morphology, avoiding cystic and necrotic areas, and with DW images, T2W images and DCE images as reference images (Fig. 2).

The mean ADC value of the tumor was calculated by averaging the ADC values of all voxels in all ROIs (Eq. 2). Because the areas of ROIs often differed greatly, the area *S*_*i*_ was the weight of the *ADC*_*i*_. The Eq. 2 could provide the mean ADC accurately. The mean ADC measured by two radiologists was definded as the final mean ADC (ADC_m_).

2 $${\text{mean ADC}} = (\sum\limits_{i = 1}^{n} ADC_{i} *S_{i})/\sum\limits_{i = 1}^{n}S_{i} $$

### Immunohistochemical analysis of Ki-67

For the immunohistochemical staining, the Ki-67 mouse monoclonal antibody (MIB-1; ZSGBBIO, Beijing, China) was used. Briefly, the paraffin Sects. (4 μm) were kept in an oven (60 °C) overnight. The sections were dewaxed in xylene (three cylinders for 10 min each), incubated with graded alcohol and then washed in distilled water for 3 times (2 min each time). The sections were repaired with citric acid antigen repairing solution, followed by immersing in 0.3% H_2_O_2_ in methanol at room temperature for 15 min. Then, the slides were washed by phosphate-buffered saline for three times (3 min each). For antigen retrieval, slides were heated in 0.01 M citrate buffer (pH 6) in an autoclave. Then, the slides were cooled to room temperature and rinsed with PBS. After nonspecific protein blockage, the slides were incubated with the primary antibody at 37 °C for 90 min. Then, the slides were incubated with corresponding secondary antibody for 20 min. Then the slides were counterstained with hematoxylin.

Ki-67 was considered positive when the cell nuclei were stained brown. The Ki-67 SI was defined as the percentage of positive nuclei out of totally 1000 cells counted according to the eyepiece grid, which was performed by a pathologist in a blinded manner.

### Statistical analysis

Statistical analysis was performed with SPSS for Windows, version 17.0 (SPSS, Chicago, Illinois). *P* < 0.05 was considered as statistically significant. Kolmogorov–smirnov test was used to detect whether the data were normally distributed. The Cho/water of type I EC was dichotomized for the analysis as Ki-67 SI ≤ 40% versus > 40% [2]. Five-year cancer-specific survival rates were 58% for those tumors with high Ki-67 expression (> 40%), compared with 88% for those with tumors with low Ki-67 expression [2]. Reliability analysis was used to test the consistency of the mean ADC between two radiologists. Comparisons of Cho/water, as well as ADC_m_, among different FIGO stages or different grades of EC were performed using one-way analysis of variance (ANOVA). The Cho/water and the ADC_m_ of type I EC with different expression levels of Ki-67 SI, with deep and superficial myometrial invasion, with and without cervical invasioin, and with and without lymph node metastasis were compared with the independent-sample t-test. The receiver operating characteristic (ROC) curve analyses was used to determine an optimal Cho/water threshold to distinguish between these two groups. The Pearson correlation test was used to analyze the correlations between the Cho/water and Ki-67 SI, between the Cho/water and tumor size, and, between the Ki-67 SI and tumor size as well as between the ADC_m_ and Ki-67 SI, and between the ADC_m_ and tumor size. The Spearman correlation test was performed to analyze the correlations between Cho/water and number of included voxels, the Ki-67 and FIGO stage, between the Ki-67 and tumor grade, between the Cho/water and FIGO stage, and, between the Cho/water and tumor grade, as well as between ADC_m_ and FIGO stage, and between the ADC_m_ and tumor grade.

## Results

### Basic clinical data

Initially, 50 cases were eligible for inclusion. Among them, 5 cases had no satisfactory MRS voxel, mainly due to the unstable baseline and FWHM greater than 15 Hz. Finally, 45 cases were enrolled. Their mean age was 56.4 ± 6.8 years old. The basic clinical data of patients were shown in Table 2. The Cho peak was observed for all 45 patients with type I EC. Our results showed that the Cho/water (*P* = 0.843), Ki-67 SI (*P* = 0.638) and tumor size (*P* = 0.889) were normally distributed.

**Table 2:** The basic clinical data of patients

Items	Number
Total	45
*Ki-67 SI*	
≤ 40%	33
> 40%	12
*Myometrial invasion*	
Superficial	30
Deep	15
*Cervical invasion*	
Yes	6
No	39
*Lymph node metastasis*	
Yes	5
No	40

*SI* Staining index

### Cho/water of EC with high and low Ki-67 SI

**Fig. 3:**
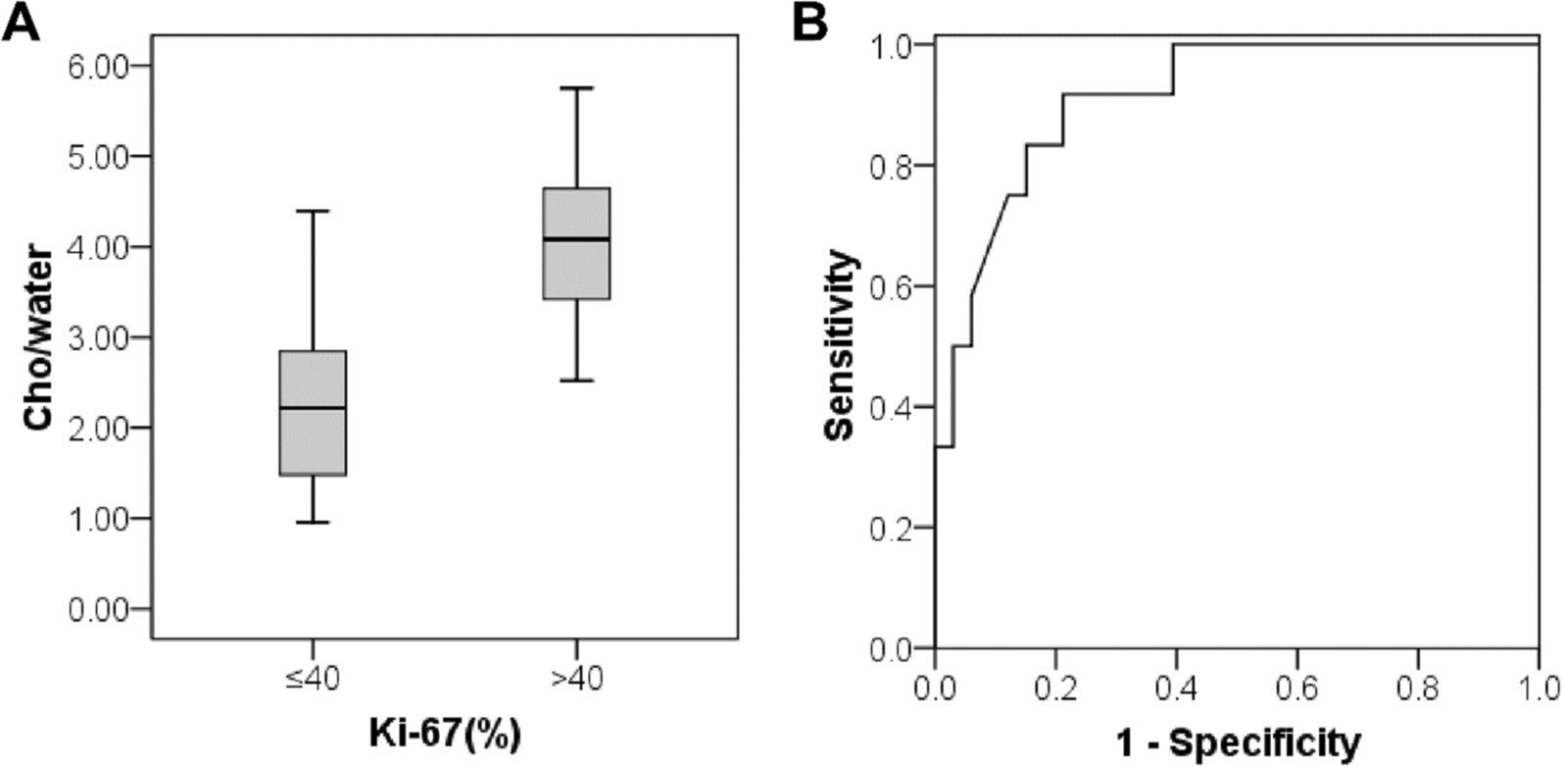
Differentiatioin of endometrial cancer (EC) with high and low Ki-67 SI using Cho/water. **A** Box plot of Cho/water (× 10^−3^) obtained in EC with Ki-67 SI ≤ 40% and > 40%. The Cho/water in EC of Ki-67 SI > 40% was significantly higher than that of Ki-67 SI ≤ 40%. **B** ROC curve was used to differentiate EC of Ki-67 SI > 40% from EC of Ki-67 SI ≤ 40%

**Fig. 4:**
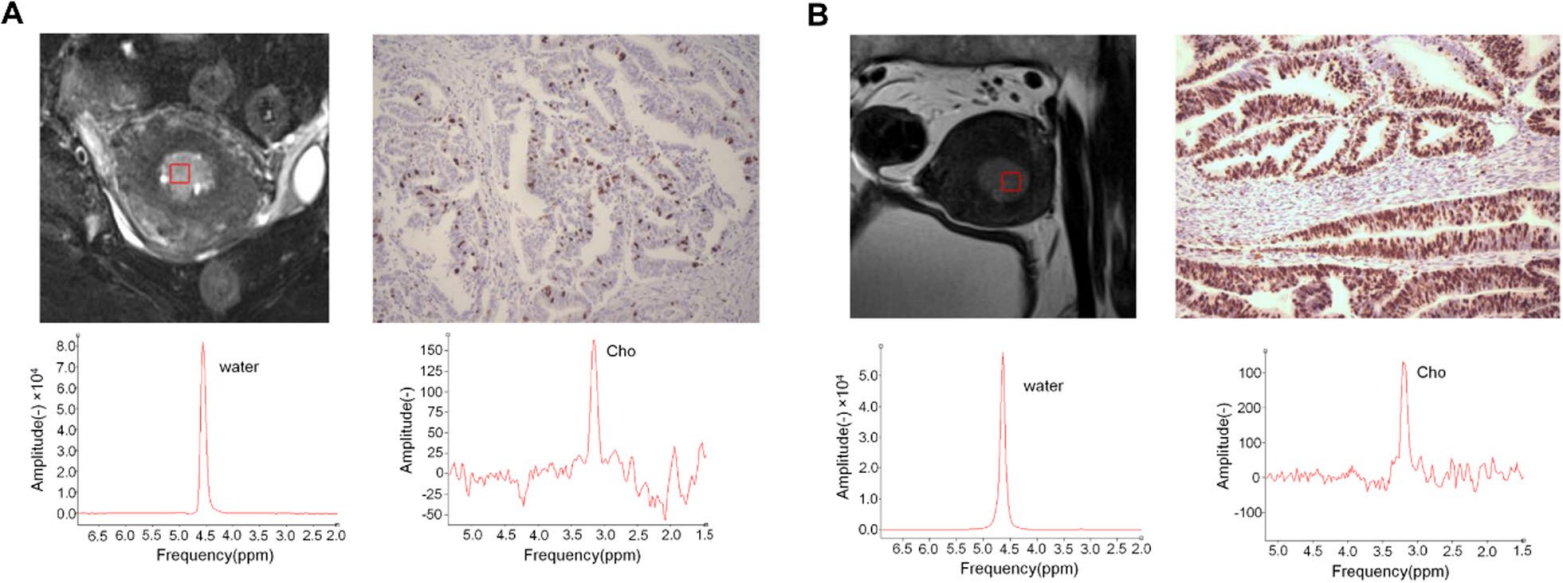
Ki-67 images and MRS of endometrial cancer (EC) patients with different level of Ki-67 SI. **A** A 51-year-old female with FIGO Ia and grade 1 EC. The Ki-67 SI was 20%. The Cho/water of the voxel was 2.03 × 10^−3^. The full widths at half maximum (FWHMs) of water and Cho peaks were 12.56 Hz and 11.43 Hz, respectively. The relative Cramér-Rao standard deviation (CRSD) of the Cho peak was about 9.7%. **B** A 55-year-old female with FIGO Ia and grade 1 EC. The Ki-67 SI was 60%. The Cho/water of the voxel was 5.47 × 10^−3^. The FWHMs of water and Cho peaks were 12.56 Hz and 11.71 Hz. The relative CRSD of the Cho peak was about 7.8%

There were 33 patients with the Ki-67 SI ≤ 40% and 12 patients with the Ki-67 SI > 40%. The mean Cho/water of the former (2.28 ± 0.93) × 10^−3^ was significantly lower than the latter (4.08 ± 1.00) × 10^−3^ (*P* < 0.001) (Fig. 3A). The area under the curve (AUC) was 0.912. The Cho/water threshold was 2.89 × 10^−3^, with the sensitivity and specificity of 0.917 and 0.788, respectively (Fig. 3B). Immunohistochemical images of EC with low and high Ki-67 SI and the corresponding Cho and water peaks were shown in Fig. 4A, B.

### Cho/water of EC with superficial and deep myometrial invasion

**Fig. 5:**
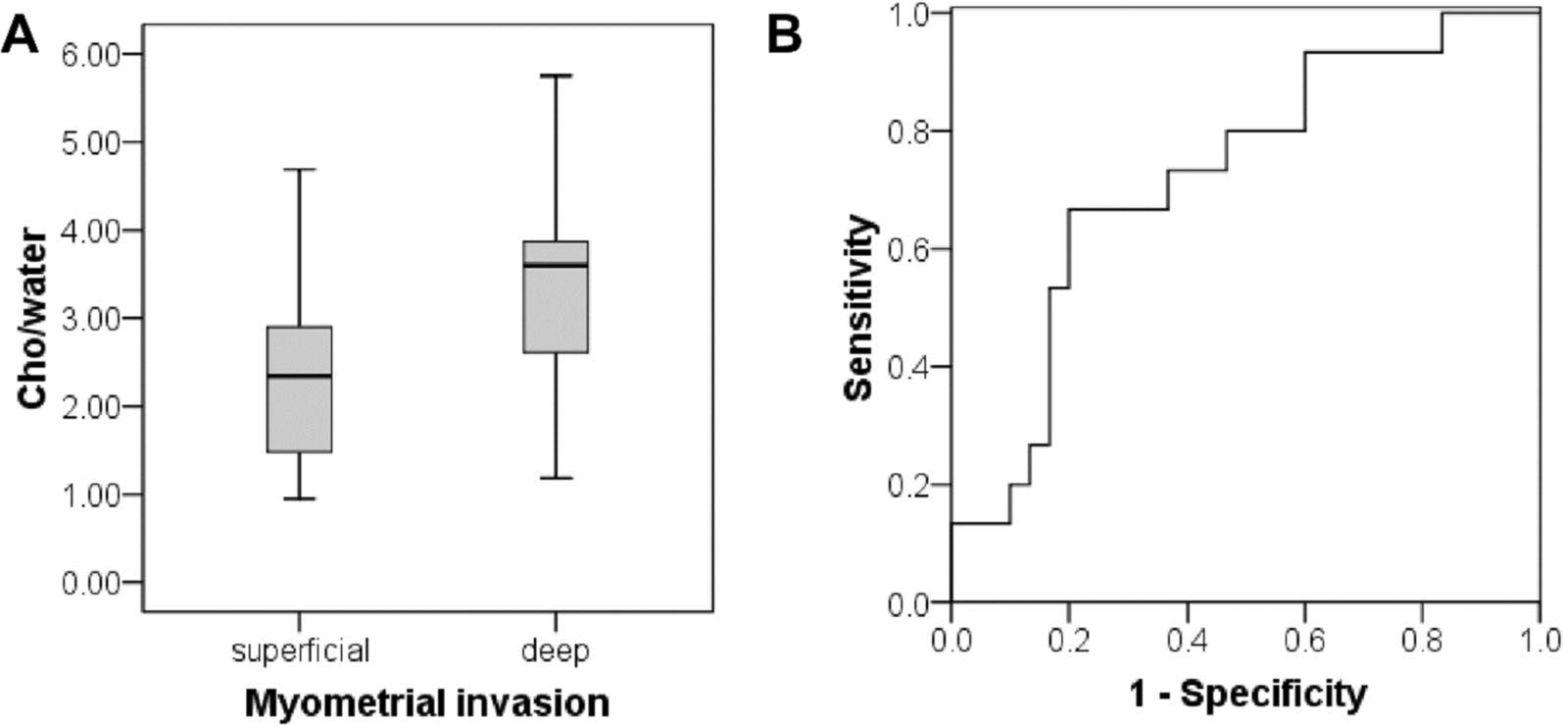
Differentiatioin of endometrial cancer (EC) with superficial and deep myometrial invasion using Cho/water. **A** Box plot of Cho/water (× 10^−3^) obtained in EC with superficial myometrial invasion and with deep myometrial invasion. The Cho/water in EC with deep myometrial invasion was significantly higher than that with superficial myometrial invasion. **B** ROC curve to differentiate EC with superficial myometrial invasion from EC with deep myometrial invasion

There were 30 patients with superficial myometrial invasion and 15 patients with deep myometrial invasion. The mean Cho/water of patients with superficial myometrial invasion (2.43 ± 1.11) × 10^−3^ was significantly lower than the patients with deep myometrial invasion (3.41 ± 1.26) × 10^−3^ (*P* = 0.011) (Fig. 5A). The AUC value was 0.722. The Cho/water threshold was 3.07 × 10^−3^, with the sensitivity and specificity of 0.667and 0.800, respectively (Fig. 5B).

### Cho/water of EC with and without cervical invaion

There were 6 patients with cervical invasion (mean Cho/water, [2.68 ± 1.00] × 10^−3^) and 39 patients without cervical invasion (mean Cho/water, [2.77 ± 1.28] × 10^−3^). There was no significant difference in the Cho/water between patients with and without cervical invasion (*P* = 0.866).

### Cho/water of EC with and without lymph node metastasis

**Fig. 6:**
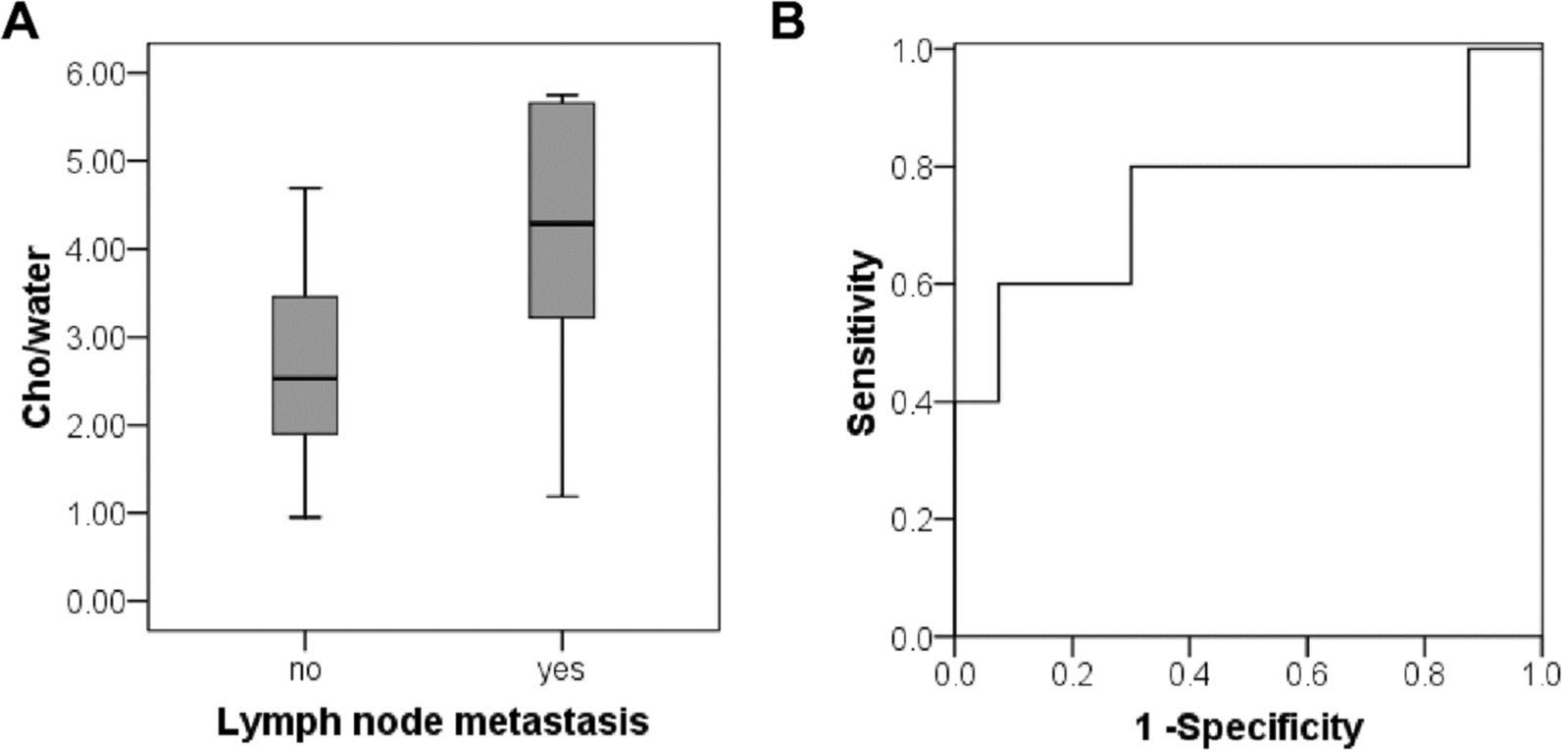
Differentiatioin of endometrial cancer (EC) with and without lymph node metastasis using Cho/water. **A** Box plot of Cho/water (× 10^−3^) obtained in EC with lymph node metastasis and without lymph node metastasis. The Cho/water in EC with lymph node metastasis was significantly higher than that without lymph node metastasis. **B** ROC curve to differentiate EC with lymph node metastasis from EC without lymph node metastasis

There were 5 patients with lymph node metastasis and 40 patients without lymph node metastasis. The mean Cho/water for patients with lymph node metastasis (4.02 ± 1.90) × 10^−3^ was significantly higher than the patients without lymph node metastasis (2.60 ± 1.06) × 10^−3^ (*P* = 0.014) (Fig. 6A). The AUC value was 0.750. The Cho/water threshold was 3.07 × 10^−3^, with the sensitivity and specificity of 0.800 and 0.700, respectively (Fig. 6B).

### Correlations between Cho/water and Ki-67, between Cho/water and size, and between Ki-67 and size of EC

**Fig. 7:**
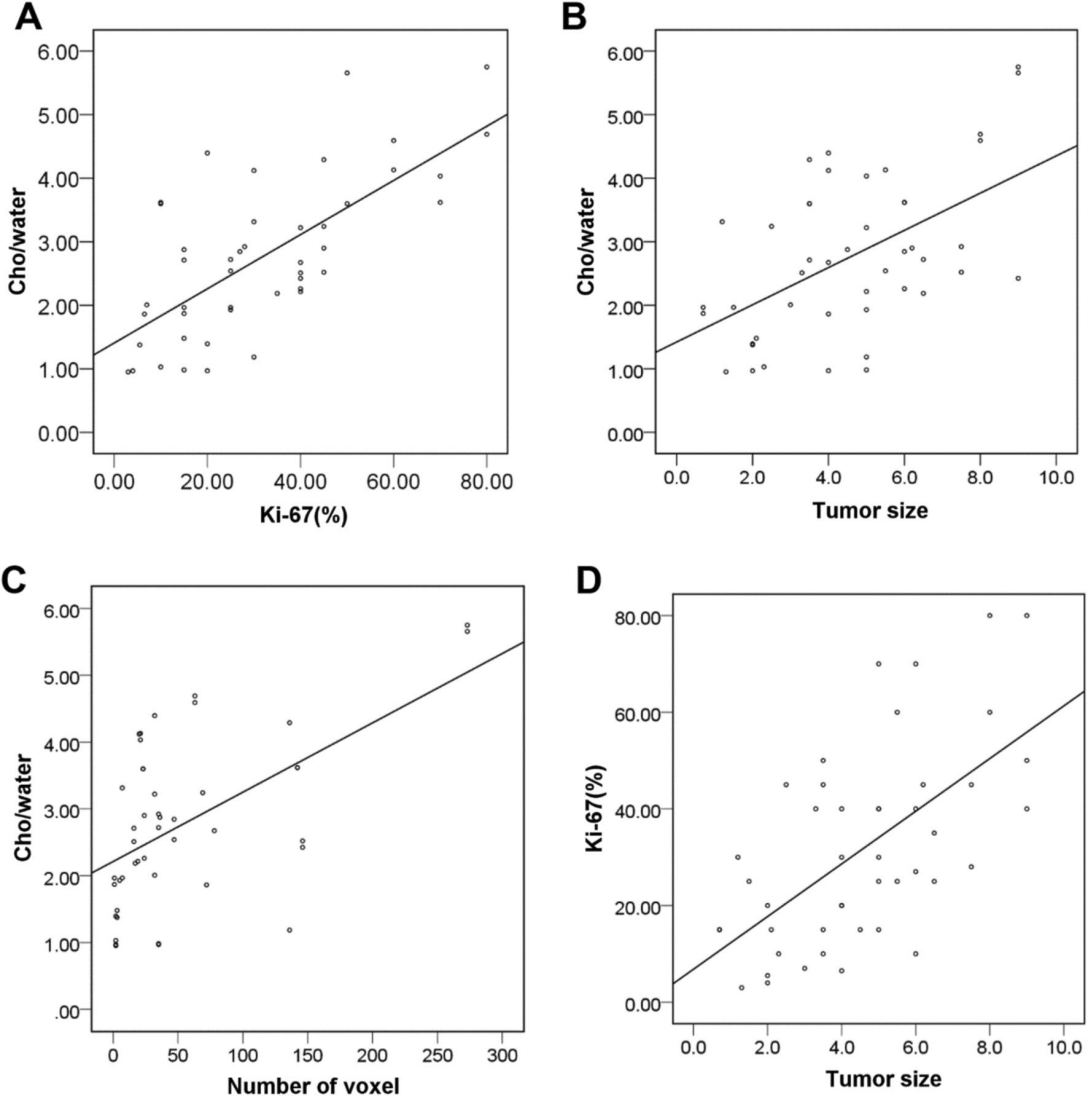
The relationship between both of Cho/water, Ki-67 and size of Type I endometrial cancer (EC). **A** Between Cho/water and Ki-67. **B** Between Cho/water and size of tumor. **C** Between Cho/water and number of voxel. **D** Between Ki-67 and size of tumor

In addition, our results showed that there was significant correlation between the Cho/water and Ki-67 SI for EC (r = 0.701, *P* < 0.001) (Fig. 7A), between Cho/water and tumor size (r = 0.538, *P* < 0.001) (Fig. 7B), between Cho/water and number of voxel (ρ = 0.500, *P* < 0.001) (Fig. 7C), and between Ki-67 SI and tumor size (r = 0.609, *P* < 0.001) (Fig. 7D).

### Correlations between the Ki-67 and FIGO stage, between the Ki-67 and tumor grade, between the Cho/water and FIGO stage, and between the Cho/water and tumor grade

The Ki-67 SI and Cho/water for EC cases of different FIGO stages and grades were shown in Table 3. Our results showed that there were no significant difference in the Ki-67 SI among different FIGO stages (*P* = 0.308). There were significant differences in the Cho/water among different FIGO stages (*P* = 0.025), but the difference only existed between FIGO Ia and III EC (*P* = 0.015). There were significant differences in the Ki-67 SI among different EC grades (*P* = 0.001). The statistical differences in Ki-67 SI existed between any two grades of EC, except between G2 and G3 EC (G1 vs. G2, *P* = 0.018; G1 vs. G3, *P* = 0.002; and G2 vs. G3, *P* = 0.248, respectively). There were significant differences in the Cho/water among different EC grades (*P* = 0.003). The statistical differences in Cho/water existed between any two grades of EC, except between G2 and G3 EC (G1 vs. G2, *P* = 0.003; G1 vs. G3, *P* = 0.040; G2 vs. G3, *P* = 0.986, respectively). The Ki-67 SI and Cho/water were positively correlated with the FIGO stage and grade (Table 3).

**Table 3:** The Ki-67 SI and Cho/water of different FIGO stages and grades of type I EC

Variable	No. of cases	Ki-67 expression (%) (Mean ± SD)		Cho/water × 10^−3^ (Mean ± SD)	
*FIGO stage*					
Ia	26	28.5 ± 20.8	^a^*P* = 0.308	2.40 ± 1.19	^b^*P* = 0.025
Ib	9	30.2 ± 22.1		2.98 ± 0.70	
II	4	34.5 ± 9.54		2.65 ± 0.22	
III	6	45.8 ± 18.6		4.04 ± 1.70	
Spearman correlation		^e^ρ = 0.295, *P* = 0.049		^f^ρ = 0.376, *P* = 0.011	
*Histologic grade*					
Grade 1	16	18.8 ± 13.6	^c^*P* = 0.001	1.95 ± 0.99	^d^*P* = 0.003
Grade 2	21	35.5 ± 19.1		3.23 ± 0.97	
Grade 3	8	47.5 ± 21.2		3.15 ± 1.58	
Spearman correlation		^g^ρ = 0.541, *P* < 0.001		^h^ρ = 0.443, *P* = 0.002	

^a^ differences in the Ki-67 SI among different FIGO stages; ^b^ differences in the Cho/water among different FIGO stages; ^c^ differences in the Ki-67 SI among different grades; ^d^ differences in the Cho/water among different grades; ^e^ the correlation coefficient between Ki-67 and FIGO stage; ^f^ the correlation coefficient between Cho/water and FIGO stage; ^g^ the correlation coefficient between Ki-67 and grade; ^h^ the correlation coefficient between Cho/water and grade

### ***The relationship between ADC***_***m***_*** and invasiveness of type I EC***

The intraclass correlation coefficient of ADC was 0.956 (*P* < 0.001) (95% confidence interval 0.922, 0.976). There were no significant differences in the tumor ADC_m_ between different levels of Ki-67 SI, between deep and superficial myometrial invasion, between with and without cervical invasioin, and between with and without lymph node metastasis (Table 4). There was no significant correlation between ADC_m_ and FIGO stage (ρ = − 0.001, *P* = 0.996), as well as between ADC_m_ and grade (ρ = − 0.237, *P* = 0.118). There was no significant correlation between ADC_m_ and Ki-67 SI (r = − 0.218, *P* = 0.151), as well as between ADC_m_ and size (r = − 0.178, *P* = 0.242).

**Table 4:** Comparisons of ADC_m_ among different groups

Invasiveness	ADC_m_ (× 10^−3^mm^2^/s)	*P* value
*Ki-67 SI*		
≤ 40%	0.873 ± 0.146	0.171
> 40%	0.810 ± 0.095	
*Myometrial invasion*		
Superficial	0.866 ± 0.153	0.531
Deep	0.838 ± 0.097	
*Cervical invasion*		
No	0.855 ± 0.146	0.843
Yes	0.867 ± 0.030	
*Lymph node metastasis*		
No	0.867 ± 0.139	0.155
Yes	0.775 ± 0.079	
*FIGO stage*		
Ia	0.864 ± 0.164	0.841
Ib	0.857 ± 0.088	
II	0.876 ± 0.033	
III	0.810 ± 0.111	
*Grade*		
G1	0.864 ± 0.126	0.065
G2	0.889 ± 0.149	
G3	0.758 ± 0.072	

## Discussion

In this study, only type I EC patients were included and investigated. The type II EC cases have multiple histopathological subtypes, such as serous carcinoma and clear cell carcinoma. Type II EC is clinically treated as a high grade of malignancy and is no longer graded in pathology. In previous studies, we differenciated the type II EC from type I EC with MRS [6, 7]. Therefore, the type II EC cases were not studied herein.

No Ki-67 expression was detected during the G0 and early G1 phase in the cell cycle. Ki-67 expression would be detected in the late G1 phase, which gradually increases in the S and G2 phases, and then rapidly degrades after peaking in the M phase [10]. Ki-67 is a key factor in ribosome synthesis, is crucial for cell proliferation and closely related to cell anabolism [11]. Cho is a marker for cell proliferation, which is often elevated in cancer and associated with tumor progression [12]. High expression of Ki-67 has been associated with reduced EC-specific survival [13]. To the best of our knowledge, there has been no reports studying the EC proliferation using MRS.

In this study, the Cho peak was observed in all type I EC cases. The Cho/water of EC with high Ki-67 SI was higher than with low Ki-67 SI. The Cho/water and Ki-67 SI were positively correlated. The Ki-67 expression reflects the proliferative activity of tumor, and Cho is a marker for cell proliferation [12]. Cell proliferation per unit volume may result in a high cell density and relative reduction of extracellular free water [14], which may result in a decrease in the water peak. This may explain the positive correlation between Cho/water and Ki-67 SI of EC. There are few studies on EC proliferation using MRS. However, there are relevant studies on proliferation of glioma and prostate cancer. These results showed that the level of Cho was positively correlated with the proliferative activity [15, 16]. The Cho/water for EC patients with deep myometrial invasion, as well as with lymph node metastasis, was significantly higher than that with superficial myometrial invasion, as well as without lymph node metastasis. This is different from previous study [17], which has shown that the differences are not statistically significant. There was no significant difference in the Cho/water between patients with and without cervical invasion, which was in line with the previous study [17]. The heterogeneity of MRS Cho/water within solid part of EC was studied in our previous study [6], which showed that the Cho/water heterogeneity of Ib and III EC was significantly greater than that of Ia EC; and the Cho/water heterogeneity of EC was increased with the increased tumor stage and size.

In this study, we found that the Cho/water was positively correlated with the tumor grade. There were statistical differences in Cho/water between any two grades of EC, except between G2 and G3 EC. This is different from the previous study, which reported that there were no significant differences among different grades of EC and no correlation between Cho/water and tumor grades [6]. The reason may be that 1) only type I EC was included in this study; 2) the number of patients with EC included in this study was more than that of the previous study. But the results were similar to those in a previous study [17]. The relationship between Cho/water or signal to noise ratio of Cho and FIGO stage was in line with the previous studies [6, 7]. In a study with large series, tumor size was demonstrated as an independent prognostic factor of local recurrence in women with low-risk EC and could be a valuable additional criterion to personalize the treatment approach to these patients [18]. In our study, the number of included voxels per patient reflected the size of tumor. Cho/water was found to be positively correlated with the number of included voxels. Ki-67 and Cho/water were positively correlated with tumor size, which is partly similar to previous studies [6, 7, 17]. Tumor tCho/Creatine ratio is reported to be positively correlated to MRI-measured tumor volume [17]. This may be also explained that both of Ki-67 and Cho/water can reflect the proliferative activity of tumor cells.

In patients with EC, endometrium without tumor proliferation would be difficult to display. The voxel size of MRS was 7 mm × 7 mm × 7 mm. Therefore, the adjacent endometrium was too small to obtain the accurate metabolites from MRS. Therefore, Cho/water of EC was not compared with that of the adjacent endometrium. In some patients, the lesions invaded the whole myometrium, and there was almost no normal myometrium, or the normal myometrium was very thin. Thus, the MRS of the normal myometrium was not studied.

In previous studies, other MR parameters or MR image texture parameters were generally used to study the correlation with Ki-67 and other invasive indicators [14, 19–24]. The relationship between Ki-67 and ADC is controversial in patients with EC. A study has shown that Ki-67 and ADC are negatively correlated [14], while another study has indicated that there is no correlation between them [22]. In this study, we also found that there was no significant correlation between ADC_m_ and Ki-67 SI. Both the DWI and DKI parameters provide valuable imaging biomarkers for EC diagnosis and the assessment of risk stratification in EC [19–22]. However, in this study, we found no significant difference in ADC_m_ among different grades of EC, as well as different FIGO stages of EC. The APT (amide proton transfer) imaging signal intensity is reported to be positively correlated with the histologic grades of endometrioid EC, but the mean and minimum ADCs shows no significant differences among the three histologic grades [23]. The combination of tumor volume ratio and ADC can be used for predicting the tumor grade, lymphovascular invasion, and depth of myometrial invasion, but there is no significant difference in the ADCs between grades 1 and 2 tumors [24]. MRI-derived tumor texture parameters could independently predict the deep myometrial invasion, high-risk histological subtype, lymphovascular space invasion, high-grade tumor and reduced survival in EC [25, 26]. Ktrans and Ve values are significantly higher in low grade but no significant correlations are found between quantitative perfusion parameters and histological type, lympho-vascular invasion, or FIGO stage [27]. Low tumor blood flow and low rate constant for contrast agent intravasation are associated with high-risk histological subtype; but the derived DCE parameters of tumor are not significantly different in patients with more advanced stage, i.e., the deep myometrial invasion, cervical stroma invation or lymph node metastases [28]. Haldorsen et al*.* [29] have reported that tumor blood flow and capillary transittime had significant impacts on recurrence-/progression-free survival; but there were no significant differences in the tumour perfusion parameters among tumour grades, stage, tumour type, tumours, with or without lymph node metastases or among tumours with or without deep myometrial invasion. Therefore, there are many studies on DWI to assess the aggressiveness of EC, but the relationship between ADC and Ki-67, as well as the relationship between ADC and tumor grade, are controversial. The role of DCE parameters in EC invasiveness varys in different studies. The relationship between the Cho/water obtained from MRS and Ki-67 SI of type I EC has been rarely studied. Herein, we found that Cho/water and Ki-67 had good correlation.

There were some limitations for this study. First, this was a retrospective study. Second, the number of patients was small, especially the patients with cervical invasion and with lymph node metastasis. Third, this was a single center study. Further in-depth studies are still needed to address these issues in the future.

## Conclusions

In conclusion, the Cho/water of type I EC was positively correlated with proliferative activity. High levels of Cho/water were associated with deep myometrial invasion, lymph node metastasis, high grade of EC, high FIGO stage of EC, and big size of tumor, but not cervical invasion. Therefore, MRS is helpful for preoperative assessment of clinicopathological features of type I EC.

## Data Availability

The datasets generated and/or analysed during the current study are not publicly available due to local ownership of the data but are available from the corresponding author on reasonable request.

## Abbreviations

MRS: Magnetic resonance spectroscopy
EC: Endometrial cancer
SI: Staining index
Cho: Choline-containing compounds
T2W: T2-weighted
DWI: Diffusion-weighted imaging
DCE: Dynamic contrast enhanced
SNR: Signal-to-noise ratio
